# Change in the H–V Interval Without a Change in QRS Morphology During Atrial Pacing in a Case of Wolff–Parkinson–White Syndrome—What is the Mechanism?

**DOI:** 10.19102/icrm.2022.130401

**Published:** 2022-04-15

**Authors:** Debabrata Bera, Indranil Dutta, Suchit Majumder, Rakesh Sarkar, Sanjeev S. Mukherjee

**Affiliations:** ^1^Department of Cardiology, Rabindranath Tagore International Institute of Cardiac Sciences (RTIICS), Kolkata, India; ^2^Department of Cardiology, Apollo Gleneagles Hospital, Kolkata, India; ^3^Department of Cardiology, Medanta the Medicity, Gurugram, India; ^4^Department of Cardiology, Medica Superspeciality, Kolkata, India

**Keywords:** Complete heart block, mechanical bumping of infra-His conduction system, variable H–V interval

## Abstract

In the background of an accessory pathway (AP), the H–V interval can vary during atrial/coronary sinus pacing, but only with a concomitant change in the QRS morphology and the degree of pre-excitation. In an interesting case of a 62-year-old woman, the H–V interval varied during coronary sinus pacing despite a fixed pre-excitation. This appears to have happened due to infra-Hisian complete atrioventricular dissociation, which resulted from iatrogenic mechanical bumping of the left anterior fascicle in the background of right bundle branch block and left posterior hemiblock.

## Case presentation

**Figure 1:**
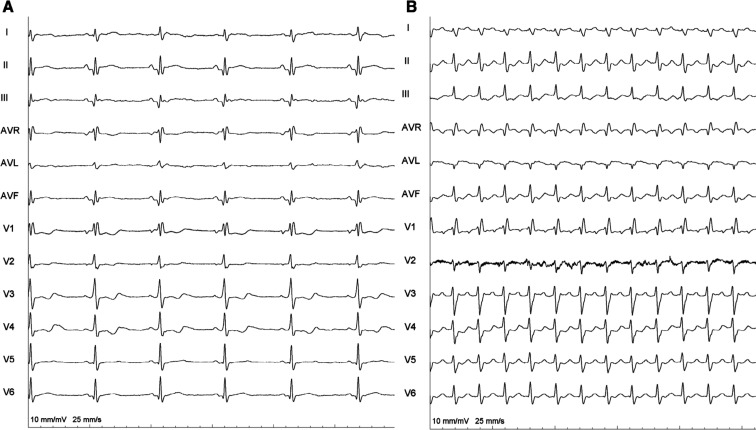
**A:** Baseline electrocardiogram showing sinus showing rhythm and right bundle branch block (RBBB). There is only subtle preexcitation, better seen in V2–V4 precordial leads. **B:** During orthodromic atrioventricular reentrant tachycardia, the underlying left posterior fascicular block was unmasked, along with the RBBB.

**Figure 2:**
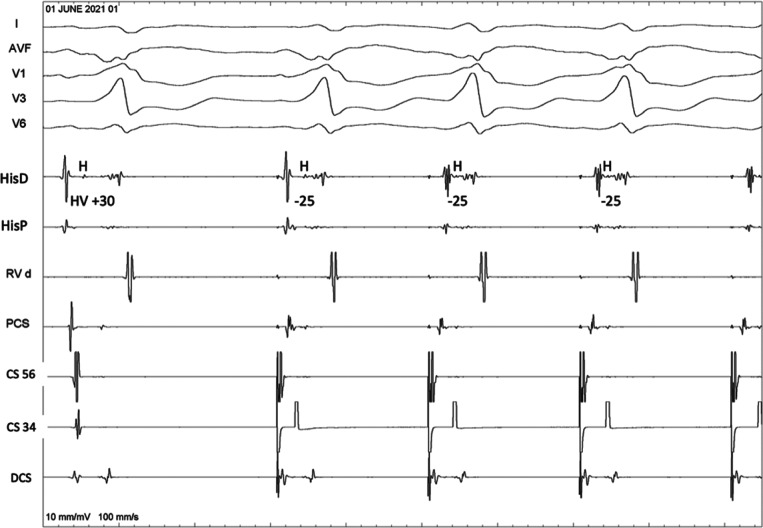
During atrial pacing from the coronary sinus 34 bipole, there was a change in the H–V interval despite the identical QRS morphology.

A 62-year-old woman with a structurally normal heart underwent an electrophysiology study (EPS) for frequent supraventricular tachycardia, having subtle pre-excitation on a baseline electrocardiogram (ECG) **(Figure 1A)**. A left-sided manifest accessory pathway (AP) was found during the EPS, and an orthodromic atrioventricular reentrant tachycardia (ORT) was easily and reproducibly induced (right bundle branch block [RBBB] morphology) **(Figure 1B)**. Mapping was attempted via the retrograde transaortic route with a small, curved, non-irrigated 4-mm-tip ablation catheter. After catheter entry into the left ventricle (LV), the pre-excitation became more evident. During an atrial pacing protocol (from the coronary sinus [CS-34]), the H–V interval shortened, but the QRS morphology remained identical **(Figure 2)**. In the background of an atrioventricular AP, the H–V interval can vary during atrial/CS pacing, but only so together with a concomitant change in the QRS morphology and degree of pre-excitation. What could be the mechanism for it?

## Discussion

**Figure 3:**
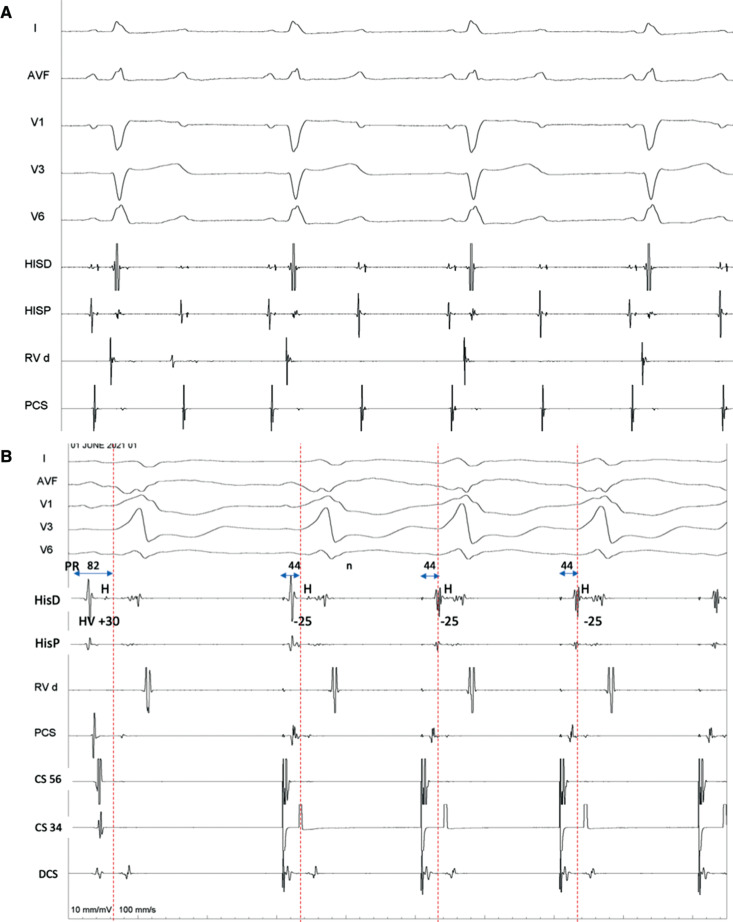
**A:** Intracardiac electrograms showing infra-Hisian complete heart block after ablation of the left lateral accessory pathway. **B:** The annotated **Figure 2** shows variable P–R and H–V intervals during coronary sinus pacing despite no change in QRS morphology (discussed in text).

**Figure 4:**
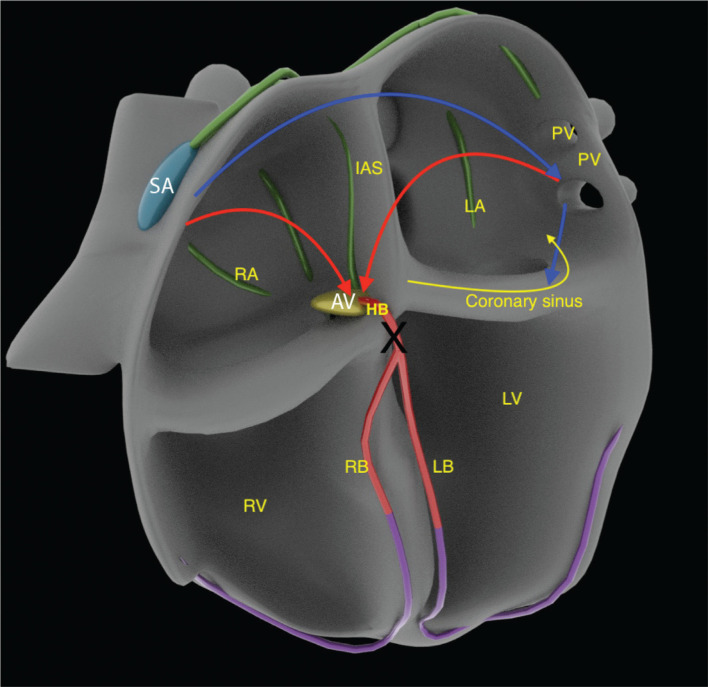
Appendix 1: Schematic diagram illustrating the mechanism of varying surface P to QRS time or P–R interval (blue arrows). The time required is much shorter from the mid–coronary sinus. The only route for A→V conduction (short blue arrow). The local A–H interval is similar from the nearly equidistant pacing site (red arrows). These, in turn, automatically result in varying H–V intervals despite an identical QRS morphology (maximum preexcitation in all beats). The black cross indicates infra-Hisian complete heart block.

Once the left lateral AP was successfully ablated, the mechanism became evident retrospectively. An infra-Hisian complete heart block (CHB) **(Figure 3A)** was unmasked, which made the above finding possible with the H–V interval changing despite having an identical QRS morphology. The fully pre-excited QRS with variable H–V interval was due to different sites of atrial stimulation (sinus node in the first beat, fusion of sinus and CS in the second beat, full CS pacing in the third beat, and so on). The different stimulation sites led to a different P to QRS onset time (P–R interval). The P–R interval shortened when pacing was performed from CS-34 (closer to the AP). However, the local A–H interval changed minimally as it was still conducting via the intact supra-His atrioventricular (AV) node, which was nearly equidistant from the sinus versus the mid-CS. These findings automatically led to the shortening of the H–V interval (−25 ms) in the paced beats **(Figure 4)**. Although the AH was still a true interval, the H–V interval was essentially artificial in the absence of any true nodal conduction. Likewise, the QRS morphology remained identical as, in the presence of CHB, there was only a single route from A→V via the AP irrespective of the pacing site **(Figure 4)** with no scope for fusion.

It was initially thought that the CHB has resulted from inadvertent “mechanical bumping” of the left bundle (LB) during the entry of the ablation catheter into the LV. This is, however, not common, as the LB is a broad structure, unlike the relatively slender right bundle branch. It was retrospectively observed that there was a pre-existing left posterior fascicular block (LPFB) along with the RBBB. The LPFB was possibly masked in the sinus rhythm by the antegrade AP conduction **(Figure 1A)**. However, as expected, it was evident during ORT **(Figure 1B)**. The ablation catheter would have bumped the slender left anterior fascicle during the LV entry, leading to the infra-Hisian CHB **(Figure 3A)**. The finding of manifest pre-excitation becoming abruptly overt after LV catheter entry also supported our speculation. In fact, the ORT also became non-inducible.

Iatrogenic bumping of the AP was suspected on the table, and it was expected that it would recover over time. Due to disabling tachycardia, a shared decision of AP ablation was planned after discussion with the patient’s relatives. As expected, CHB ensued as soon as the AP conduction was ended, but the antegrade conduction did not recover over the next 90 minutes, and a temporary pacemaker was placed. Unexpectedly, the AV nodal conduction did not recover over the next 36 hours, and a permanent pacemaker had to be implanted. It is also possible that the patient had had intermittent CHB earlier but was protected from bradycardia symptoms by the antegrade conduction of the AP until ablation. During follow-up, her AV conduction was noted to be restored, but she still required 9% RV pacing. This interesting case highlights the importance of careful analysis of sinus and tachycardia ECGs to obtain an exact picture of nodal and infra-Hisian conduction systems, even in cases ablated laterally far away from the AV node. Retrospectively, it appears that mapping and ablation via a trans-septal approach might be safer in patients with RBBB to avoid an iatrogenic AV block due to bumping.

